# Whole genome sequencing reveals widespread distribution of typhoidal toxin genes and VirB/D4 plasmids in bovine-associated nontyphoidal *Salmonella*

**DOI:** 10.1038/s41598-018-28169-4

**Published:** 2018-06-29

**Authors:** Enrique Jesús Delgado-Suárez, Nelly Selem-Mojica, Rocío Ortiz-López, Wondwossen A. Gebreyes, Marc W. Allard, Francisco Barona-Gómez, María Salud Rubio-Lozano

**Affiliations:** 1 Facultad de Medicina Veterinaria y Zootecnia, Universidad Nacional Autónoma de México, Mexico City, 04510 Mexico; 2 Evolution of Metabolic Diversity Laboratory, Unidad de Genómica Avanzada (Langebio), Cinvestav-IPN, Irapuato, 36821 Mexico; 3 Centro de Investigación y Desarrollo en Ciencias de la Salud, Universidad Autónoma de Nuevo León, Monterrey, 66460 Mexico; 4 College of Veterinary Medicine, The Ohio State University, Columbus, 43210 USA; 5 Office of Regulatory Science, Center for Food Safety and Applied Nutrition, U. S. Food and Drug Administration, College Park, 20740 USA; 6 Present Address: Tecnológico de Monterrey, School of Medicine and Health Sciences, Monterrey, 64710 Mexico

## Abstract

Nontyphoidal *Salmonella* (NTS) is a common pathogen in food-producing animals and a public health concern worldwide. Various NTS serovars may be present in apparently healthy animals. This could result in carcass contamination during the slaughter process leading to human exposure. While most genomic research has focused on *Salmonella* pathogenesis, little is known on the factors associated with subclinical infections and environmental persistence. We report here the widespread distribution of typhoidal toxin genes (i. e. the *cdtB* islet, *hlyE*, *taiA*), among NTS strains from a beef slaughter operation (n = 39) and from epidemiologically unconnected ground beef (n = 20). These genes were present in 76% of the strains, regardless of serovar, isolation source or geographical location. Moreover, strains that predominated in the slaughterhouse carry plasmid-borne type IV secretion systems (T4SS), which have been linked to persistent infections in numerous pathogens. Population genomics supports clonal dissemination of NTS along the food production chain, highlighting its role as reservoir of genetic variability in the environment. Overall, the study provides a thorough characterization of serovar diversity and genomic features of beef-associated NTS in Mexico. Furthermore, it reveals how common genetic factors could partially explain the emergence and persistence of certain NTS serovars in the beef industry.

## Introduction

Nontyphoidal salmonellosis is one of the leading causes of foodborne illness around the world^1^. Moreover, its estimated cost reaches 3.3 billion US dollars per year in the United States of America (USA) alone^2^. Despite the application of control strategies, food-producing animals continue to be a significant contributor to disease burden in humans^3^. However, there is limited information on why certain *Salmonella* strains colonize the intestines of livestock without causing apparent illness in the host, creating a reservoir for human diseases^4^. Hence, understanding the factors behind NTS resilience is of utmost importance from a public health perspective.

Extensive research with *Salmonella enterica* subsp. *enterica* serovar Typhimurium has revealed some virulence factors that are involved in the intestinal colonization of major livestock species^5^. However, comparative genomic analyses showed that there is considerable variation in virulence genes across serovars^6^. In spite of this, experimental evidence, particularly in beef-associated isolates, deals primarily with a small number of virulence genes, as well as epidemiological typing of isolates based on pulsed-field gel electrophoresis (PFGE)^7,8^. Conversely, genome-wide analyses have not been fully explored, except for a limited number of studies focusing on locally-relevant serovars^9,10^. Therefore, our aim is to generate a virulence genomic profile and identify genetic differences that may be related with host adaptation and overall pathogenicity in NTS associated with beef cattle.

**Figure 1:**
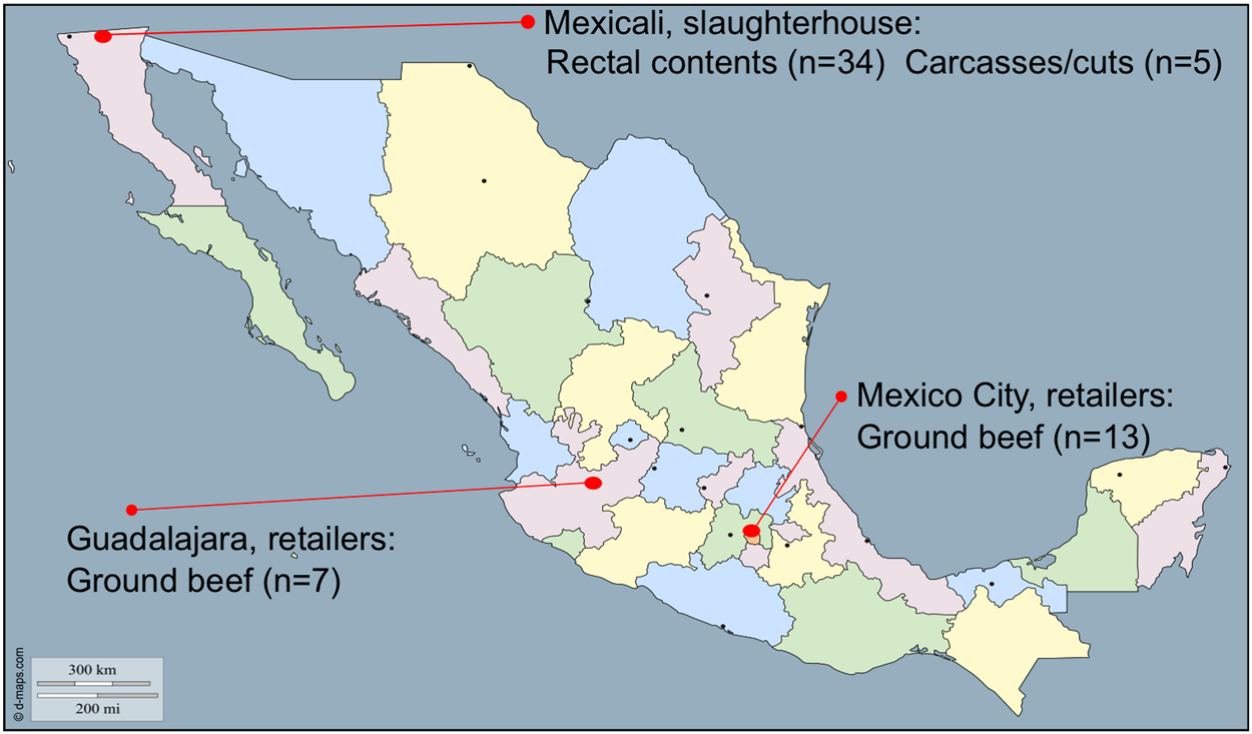
Number of NTS isolates collected by geographical location and step of the beef production continuum.

Here, we set up a long-term surveillance experimental scheme to conduct comparative genomics of bovine-associated *Salmonella* strains isolated from non-clinical sources along the beef production continuum in Mexico. We used a panel of 59 NTS isolates from previous studies conducted from March through November 2013 by our research group (Fig. 1). Our collection includes strains from multiple serovars and distant geographical regions within Mexico. We assessed their genetic diversity and conducted comparative genomics of virulence factors, stress response genes and plasmids, with the finding that most strains carry a conserved repertoire of virulence and stress response genes. Additionally, over 76% of isolates carry typhoidal toxins, while strains of the overrepresented *S. enterica* subsp. *enterica* serovar Montevideo were the only ones carrying plasmid-borne T4SS. This study provides evidence of clonal dissemination of NTS along the food production chain, highlighting the significance of isolates colonizing apparently healthy animals, as reservoirs of genetic variability in the environment. Further research is needed to test whether the observed differences affect host adaptation and/or pathogen fitness under intensive beef cattle production settings.

## Results

### Population genomic analysis shows clonal dissemination of NTS along the food production chain and limited intraserovar variation

*S*. Montevideo was overrepresented in the slaughterhouse (n = 29), accounting for nearly 75% of the total number of isolates from faeces, carcasses, and beef cuts. Among ground beef isolates, *S. enterica* subsp. *enterica* serovar Give was the most frequently (8/20) found. Overall, *S. enterica* subsp*. enterica* ser. Montevideo, Give, and Muenster, accounted for more than 70% of the isolated strains.

**Figure 2:**
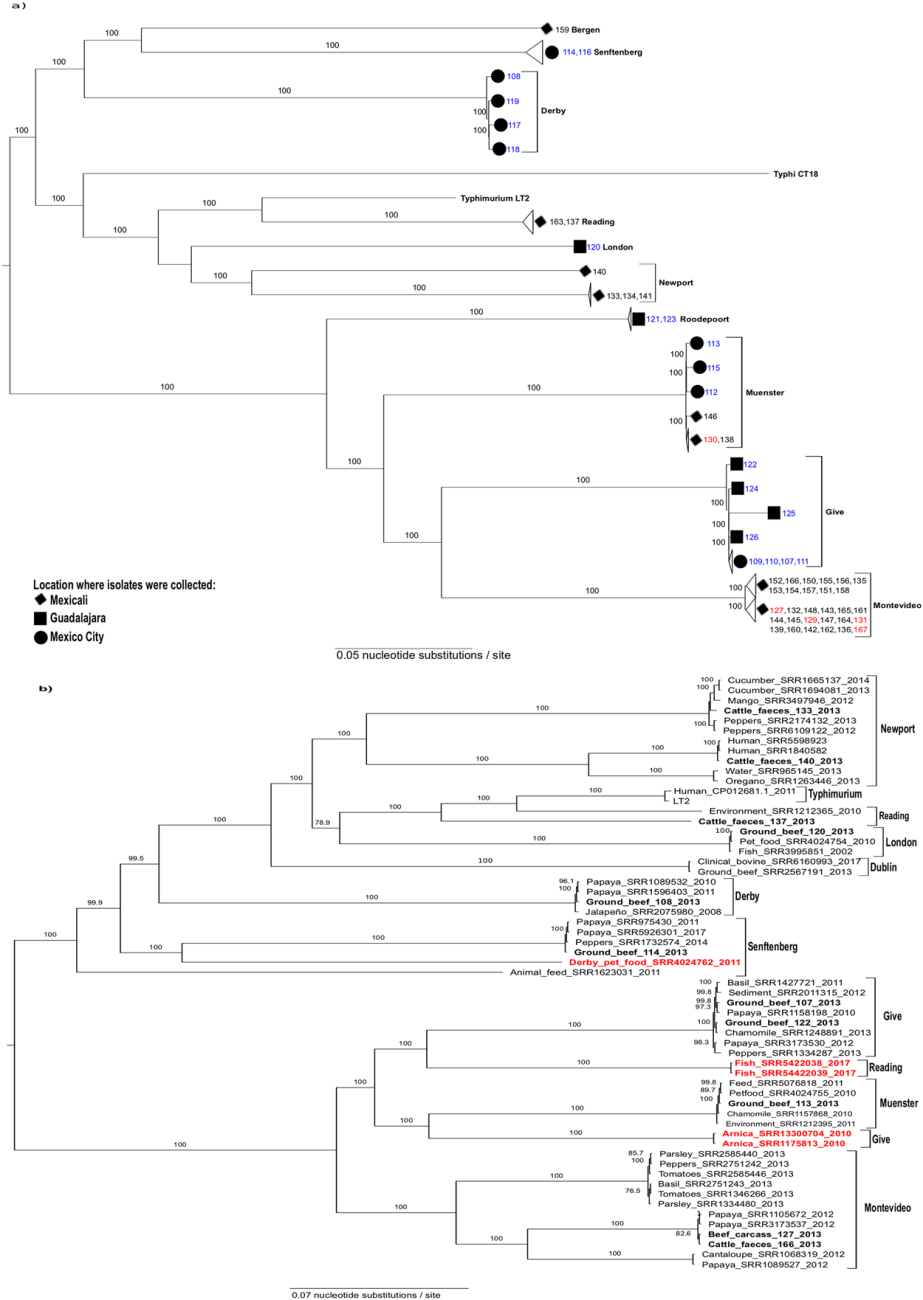
Maximum likelihood (ML) midpoint-rooted tree based on SNP analysis of: (**a**) 59 newly sequenced NTS strains, *Salmonella* Typhimurium LT2 and *Salmonella* Typhi CT18. Serovars are indicated in bold letters. Sample names are colour-coded according to isolation source (blue, ground beef; red, carcasses/cuts; black, faeces). The cities where isolates were collected are mapped onto the tree. The ML tree was generated in RAxML 7.7.1^63^ under the GTR+ Γ model of nucleotide evolution, visualized using FigTree 1.4.3, and edited with Inkscape 0.91. The best tree was estimated by RAxML rapid bootstrapping (100 iterations) and subsequent ML search. Clade support is indicated above or next to each branch as bootstrap values, except when <70%. (**b**) 11 representative strains from this study, 47 additional isolates from produce, pet food, seafood, the environment, and clinical cases within Mexico, and 2 isolates of *Salmonella enterica* ser. Dublin. Isolation sources, NCBI accessions and/or sample names, and collection dates of isolates (if reported) are shown at tip labels. Isolates from this study are highlighted with bold letters. Serovars are mapped onto the tree. Isolates clustering with those of a different serovar are highlighted in red. *Salmonella* Typhimurium LT2 was used as a reference. The ML tree generation procedure and statistical support are the same as indicated in **a**.

According to genome annotation, isolates of the same serovar represent the same multi-locus sequence type (ST), except those of *S. enterica* subsp. *enterica* ser. Give and Newport (see Supplementary Table S1). The STs found in strains of *S*. Newport (ST118 and ST132) are the only ones that have been frequently associated with human infections^11^. This is consistent with results of phylogenetic analysis (Fig. 2a), which broadly divided isolates into two genetically divergent sublineages with 100% bootstrap support. One sublineage was composed of *S. enterica* subsp. *enterica* serovars Reading, Newport, London, Bergen, Senftenberg and Derby isolates, which are relatively close to the classical virulent strains, represented by *S*. Typhimurium LT2 and *S*. Typhi CT18. The other sublineage includes over 76% of all isolates (45/59) from *S*. Give, *S*. Montevideo, *S*. Muenster, and *S. enterica* subsp. *enterica* serovar Roodepoort.

The phylogeny also shows isolates of the same serovar generally clustered together in well-supported clades, despite originating from different sources or sampling sites. For instance, isolates of *S*. Montevideo and *S*. Muenster from faeces, carcasses and cuts formed single clusters within each serovar, which demonstrates the faecal origin of these strains (Fig. 2a). Likewise, isolates of *S*. Muenster and *S*. Give formed closely related subclusters, despite originating from different sampling sites and/or sources. This pattern of genetic relatedness provides evidence of NTS dissemination along the beef production chain. At the same time, it raises the question of whether our bovine-associated isolates are any different from those isolated from other sources within Mexico.

To look into these possibilities in more detail, we selected representative isolates from each of our serovar subclusters, as well as additional isolates of the same serovar, from public databases, collected from produce, pet food, seafood, the environment, and clinical cases (n = 47), to conduct further phylogenetic analyses (Fig. 2b). Two isolates of *S. enterica* subsp. *enterica* serovar Dublin, which is highly adapted to bovines, were also included in the analysis. We did not include isolates of *S. enterica* serovars Bergen and Roodepoort since no strains of these two serovars were available for comparison.

The obtained phylogeny was very similar to that of bovine-associated isolates alone, albeit it showed a greater genetic diversity, as could be expected considering the wider range of NTS sources involved. Again, two well-supported divergent sublineages were identified, with one of them containing isolates (serovars Reading, London, Newport, Derby, and Senftenberg) that are closely related to virulent strains. There were two atypical *S. enterica* ser. Reading isolates that clustered in a different sublineage than their counterparts from bovine faeces and the environment. Likewise, two isolates of *S*. Give and one of *S*. Derby are closer to isolates of serovars Muenster and Senftenberg, respectively, than they are to their equivalents. The serovar of these atypical isolates was confirmed with SeqSero^12^, providing evidence of intraserovar divergence in NTS from different environmental niches.

Interestingly, the analysis also showed our bovine-associated isolates are highly clonal with isolates from other sources. For instance, our *S*. Montevideo strains are very closely related to their counterparts isolated from papaya. Likewise, our isolates of *S*. Derby, *S*. Give, *S*. Muenster, *S*. Senftenberg, and *S*. Newport formed single clusters with those collected from produce, pet food, and the environment. In all serovar subclusters, there were isolates collected in different years. Moreover, our isolates of *S*. Newport, *S*. London, and *S*. Reading are genetically closer to strains that have been involved in human infections, as well as to strains of *S*. Dublin, which causes severe enteritis and systemic infections in cattle.

Taken together, these findings provide evidence of dissemination and persistence of NTS along the food production chain. To gain insights into the potential health risks and lifestyle dynamics allowing *S. enterica* serovars from bovine reservoirs to colonize humans, we then focused on functional traits that may be revealed after comparative genomics analyses.

### Most strains have a conserved repertoire of virulence and stress response genes

Our strain collection showed limited variation in major virulence factors, stress response genes, and *Salmonella* pathogenicity islands (SPIs) 1 through 5. For instance, long polar fimbriae genes (*lpfABCDE*), which mediate selective adhesion of *Salmonella* Typhimurium to murine ileal Peyer’s patches^13^, are present only in isolates of *S*. Bergen (1/1), *S*. London (1/1), *S*. Newport (4/4), *S*. Reading (2/2), and *S*. Senftenberg (2/2). Moreover, *sodCI*, a gene responsible for protecting the pathogen from the host oxidative burst^14^, was present only in *S*. Newport isolates (4/4). The remaining strains (n = 55) carried *sodCII*, which protects the cell from exogenous oxidative damage in the extracellular environment^15^ and has <60% identity at amino acid level to *sodCI*.

**Figure 3:**
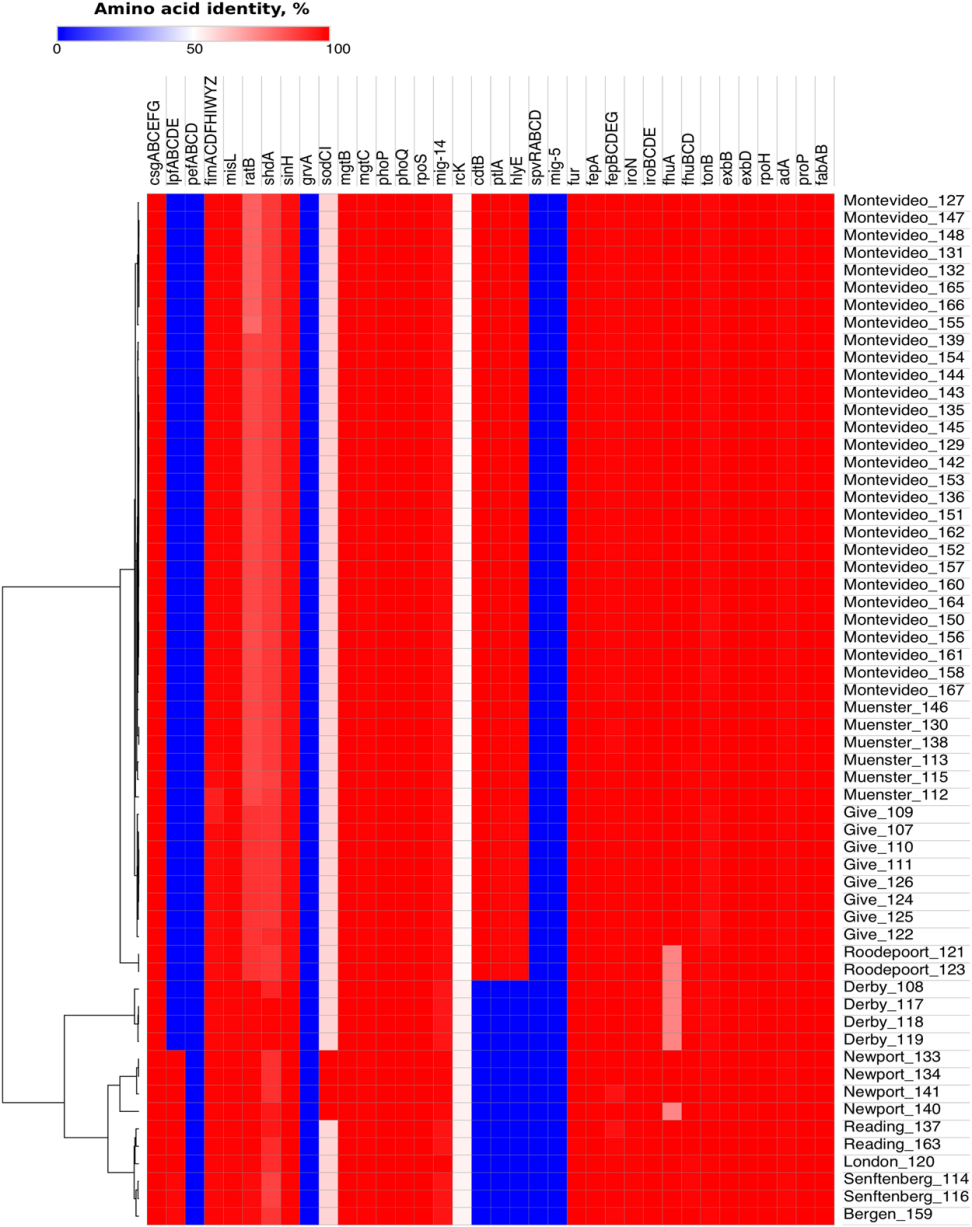
Genomic profile of major virulence and stress response factors of 59 NTS strains of different serovars. Genes are colour coded in the heat map according to amino acid similarity to the reference proteins in the virulence factors database^65^. For stress response genes, *Salmonella* Typhimurium LT2 was used as a reference. The tree on the left side shows the clustering of isolates based on average Euclidean distance. Isolates are identified by serovar and short name. Refer to Table 2 and Supplementary Table S4 for isolation source and accession numbers.

Genes that were highly conserved (≥90% amino acid similarity) included type 1 (*fimACDFHIWYZ*) and thin aggregative (*csgABCEFG*) fimbrial operons, as well as non-fimbrial adhesion factors *misL* and *sinH*, which are required for intestinal colonization^16^. Conservation was also high (95–100% amino acid similarity) in genes that take part in the activation and/or regulation of *Salmonella* invasion and intracellular survival mechanisms (*phoPQ*, *fur*, *mgtBC*, *rpoS, mig-14*), as well as iron acquisition (*iroN, fepA, fhuA*) and metabolism genes (*iroBCDE, fepBCDEG, fhuBCD, exbBD, and tonB*). The same was observed for stress response genes, which had 98–100% amino acid similarity across isolates. This included heat shock (*rpoH*), acid tolerance response (*rpoS*, *adA*), desiccation stress (*proP*) and fatty acid-associated osmo-tolerance (*fabAB*) genes. None of the isolates carried genes associated with highly virulent strains, such as those of the *Salmonella* virulence plasmid (*spvRABCD*, *pefABCD, rcK* and *mig-5*). However, all isolates carry *pagC*, which is 51% similar to *rcK* at protein level and confers serum resistance as well. A heatmap showing the virulence and stress response gene profile of the studied strains is presented in Fig. 3.

**Figure 4:**
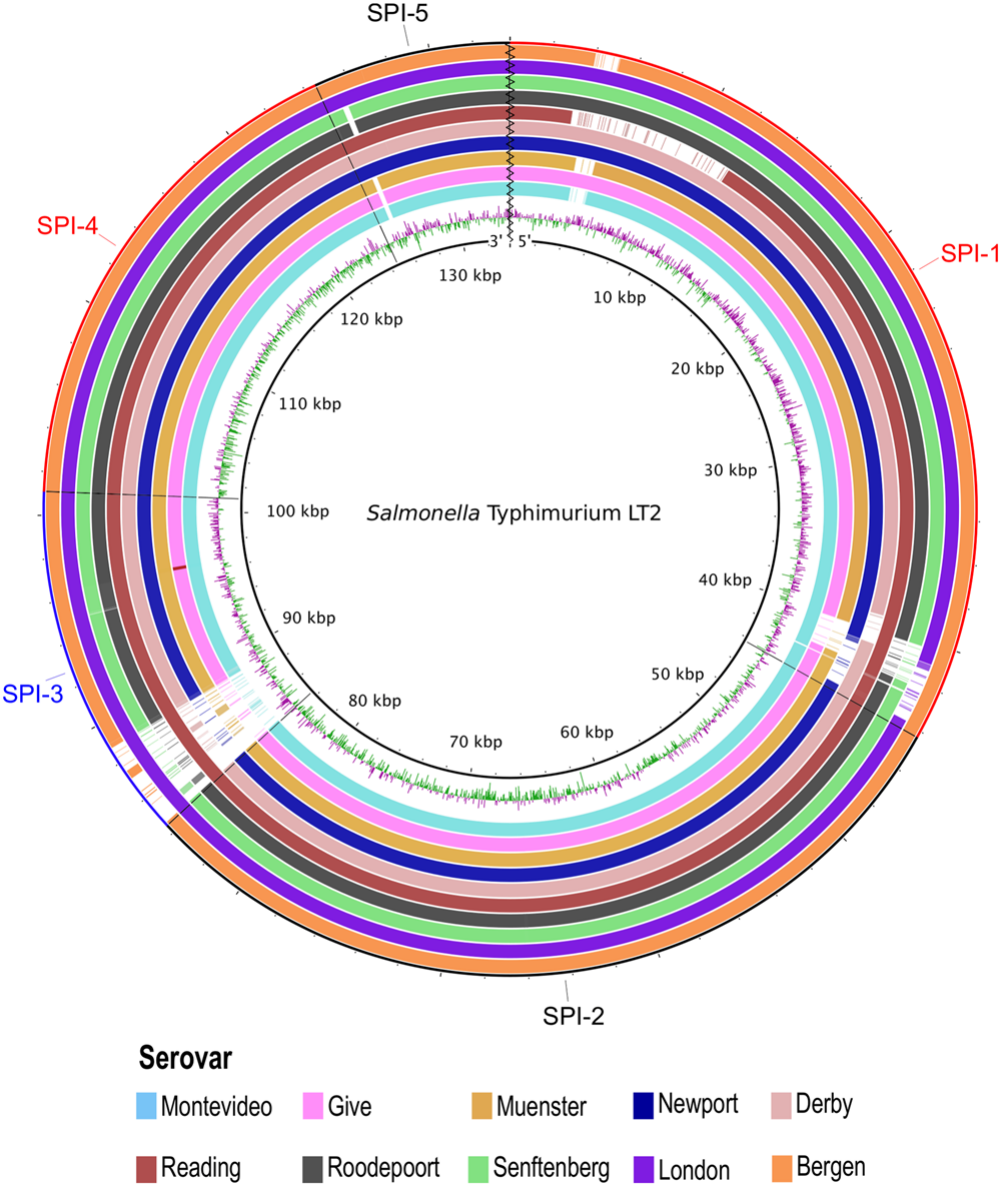
Analysis of *Salmonella* SPIs 1 to 5. The inner black circle corresponds to the reference strain Typhimurium LT2, followed by GC skew [(G − C)/(G + C)]. Regions of GC content above average are drawn in green, whereas regions below average are in purple. The remaining slots represents (from inner to outer rings) serovars Montevideo (n = 29), Give (n = 8), Muenster (n = 6), Newport (n = 4), Derby (n = 4), Reading, Roodepoort and Senftenberg (n = 2 each), London and Bergen (n = 1 each). Within each slot, the higher the colour intensity, the higher the nucleotide similarity to the reference strain.

Regarding SPIs, their nucleotide and amino acid composition was very uniform within serovars. Hence, results are reported per serovar instead of per individual isolate. There were different versions of SPIs 1 and 3, with partial deletions close to the 5′ region of both SPIs, as well as in the 3′ region of SPI-1 (Fig. 4). Conversely, SPIs 2 and 4 were 100% conserved, while the 5′ region of SPI-5 had a minor deletion in isolates of *S*. Montevideo, *S*. Give, *S*. Muenster, *S*. Roodepoort and *S*. Senftenberg, corresponding to small hypothetical proteins of unknown function.

To further characterize within-SPI variations, we conducted a sequence analysis of the encoded proteins of SPIs 1 and 3, identified in the newly sequenced isolates, against those of the LT2 reference strain. In SPI-1, the deletion on the 5′ region corresponded to the type III secretion system (T3SS) effector gene *avrA*, which inhibits cell death during the first phase of the infection^17^. Strains of *S*. Montevideo, *S*. Muenster, *S*. Reading, and *S*. Bergen lack this gene (see Supplemental Fig. S1). In addition, the iron transporter encoding gene *sitD* was truncated (73 out of 282 amino acids) in *S*. Reading isolates, due to a premature stop codon. The truncated protein is still recognized as an ABC-type Mn^2+^/Zn^2+^ transport system component through a conserved domain (CD) search against the CD database^18^. However, further review of the structure of the reference protein (H9L416) in Uniprot showed the untranslated fragment contains the functional domain, as well as 6 out 8 transmembrane-spanning regions. Therefore, most likely the truncated *sitD* gene is not encoding a fully functional protein. Isolates of *S*. Reading also had a large deletion (from *sprB* through *prgH*) that encodes several regulatory proteins and components of the T3SS apparatus. All other proteins were generally conserved, with an identity percentage ≥96% across proteins and serovars. The 3′ region of SPI-1, which had partial deletions in most strains, encodes hypothetical proteins of unknown function that are located beyond *invH*, the last gene of the invasion locus. Furthermore, the analysis of SPI-3 revealed that three genes of its 5′ region (*sugR*, STM3754, and *rhum*) were deleted in all strains but those of *S*. Reading, *S*. Senftenberg, and *S*. London (Supplemental Fig. S2). In their place, there were small insertions coding hypothetical proteins and transposases. The remaining proteins were highly conserved, with >90% amino acid similarity to those present in *Salmonella* Typhimurium LT2.

### Widespread dissemination of highly conserved typhoidal toxins among NTS isolates from the predominant sublineage

Over 76% of isolates, encompassing Montevideo, Give, Muenster, and Roodepoort serovars, carry several toxin-encoding genes. Five of these genes are part of the *cdtB* islet (SPI-11), a recognized virulence factor of *Salmonella* Typhi^19^ that activates cells’ DNA damage response, leading to cell-cycle arrest and eventually cell death by apoptosis. The key proteins encoded in this islet are PltA, PltB, and CdtB, which form the so called “typhoid toxin”. Two additional typhoidal genes, which are harboured in SPI-18 of *Salmonella* Typhi CT18^20^, were also detected in isolates carrying SPI-11. These included *hlyE*, encoding a pore-forming cytolysin, and *taiA (sty1499)*, which encodes a Typhi-associated invasin. The genomic context of both SPI-11 and SPI-18 in the newly sequenced isolates is very similar to that of *Salmonella* Typhi CT18 (see Supplementary Figs S3 and S4).

**Figure 5:**
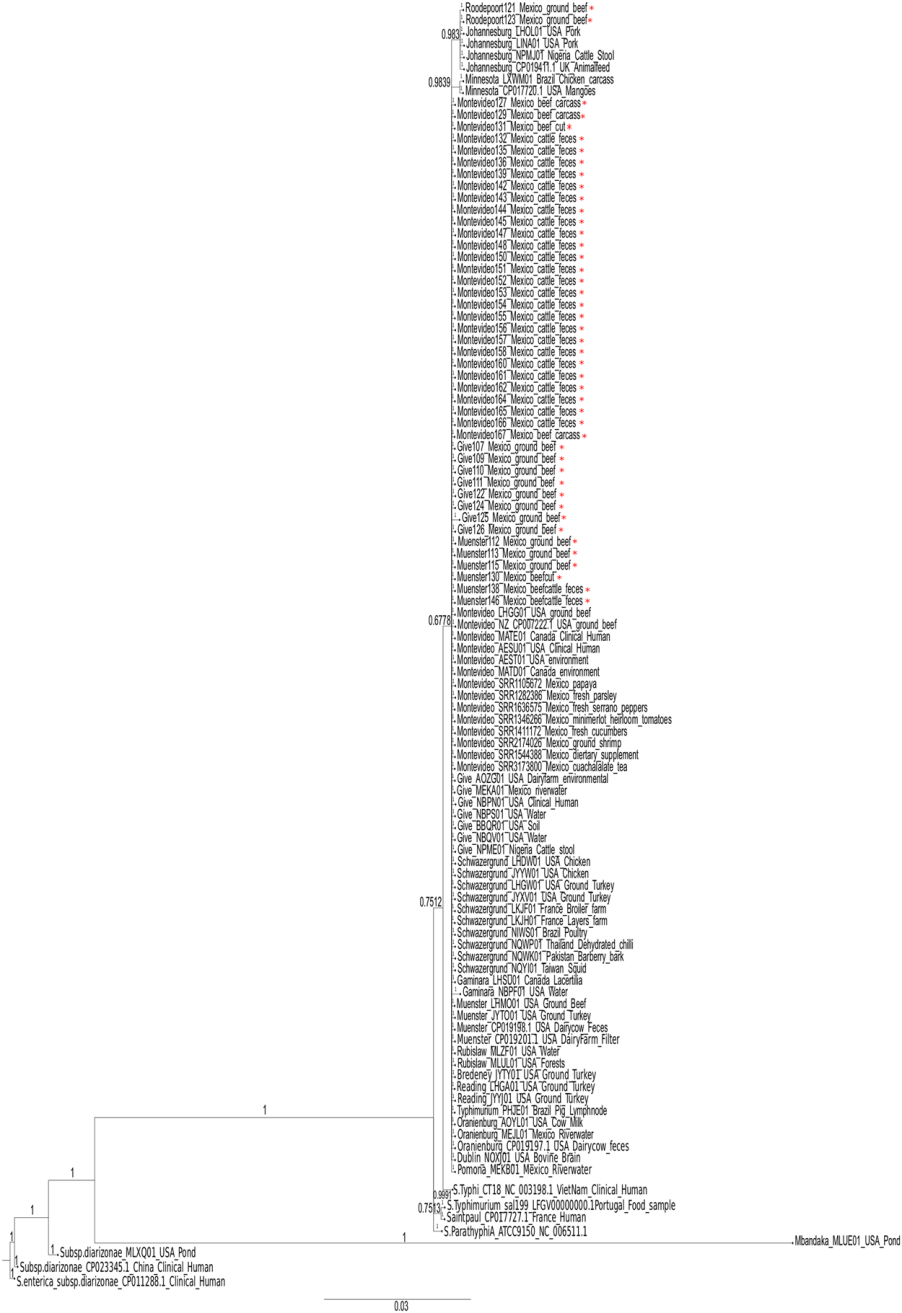
Concatenated phylogenetic analysis of CdtB, PltA, and HlyE of 59 NTS strains of different serovars and another 63 isolates available at NCBI. Posterior probabilities are indicated close to each branch. Serovar, sample name and/or accession, source, and geographical location of isolates are indicated at tip level. Samples from this study are highlighted with an asterisk. The scale indicates the number of amino acid substitutions per site. Analysis conducted with MrBayes 3.2.6^73^ in 1 million generations. For accession numbers of strains see Supplementary Table S4.

Both SPIs (11 and 18) have been reported previously in a limited number of NTS strains^21,22^. Since SPIs are thought to be associated with niche specialization, we conducted a broader phylogenetic analysis with isolates of diverse serovars, sources and regions around the world. These included strains from this study carrying SPIs 11 and 18 (n = 45), as well as another 63 publicly available isolates from four different continents (Fig. 5). The criteria used for the inclusion of isolates in the analysis are described in the methods section.

Results show SPIs 11 and 18 are highly conserved across both typhoidal and NTS isolates of different sources and countries, as indicated by the lower posterior probabilities supporting *S. enterica* subsp. *enterica* subclades. Divergence was only observed in strains of subsp. *diarizonae* and the *S. enterica* subsp. *enterica* serovar Mbandaka strain, isolated from a pond in the United States. Hence, there is no signal pointing at the modification of these genes in NTS as an adaptive response to any particular host or niche. However, their broad conservation and the low number of amino acid substitutions per site suggest fitness advantages associated with these features exceed fitness costs.

### The identified plasmids carry resistance genes, class-1 integrons, and T4SS

Overall, most plasmids encode resistance genes to both antimicrobials and heavy metals, and therefore we proceeded to investigate if these were the result of selective pressures in our system using comparative genomics. We detected replicons from eight different plasmids across strains (Table 1). The full report of plasmid prediction statistics is provided as Supplementary Information (Table S2). Likewise, the alignment of each reference plasmid to the newly sequenced isolates is provided in Supplemental Fig. S5 through S11.

**Table 1:** General features and accession numbers of the candidate plasmids identified with PlasmidFinder^a^ per *Salmonella* serovar.

Plasmid profile	Incompatibility group	Size, bp	#ORFs	NCBI Accession	Serovar (n, %)^b^
pOLA52	IncX1	51,602	68	EU370913	Montevideo (18, 62)
pIGJC156	—	5,146	3	NC-009781	Montevideo (14, 48)
IncFII(p96A)	IncFII	67,727	102	JQ418521	Montevideo (11, 38)
pK245	IncR	98,264	90	DQ449578	Muenster (6, 100) Senftenberg (2, 100)
pNDM-KN	IncA/C2	162,746	137	JN157804	Give (7, 88) Reading (2, 100)
R478	IncHI2A	274,762	295	BX664015	Give (7, 88)
pRSF1010_SL1344	IncQ1	8,688	12	HE654726	London (1, 100)
R27	IncHI1A	180,461	207	AF250878	Give (1, 13)

^a^PlasmidFinder 1.3^74^. Results based on raw reads and an identity threshold of 95%.

^b^Number and percentage of isolates of the same serovar carrying the candidate plasmids.

The R478 (IncHI2A) plasmid, predicted in *S*. Give strains, encodes chloramphenicol (*cat*), tetracycline (*tetA*) and aminoglycoside (*aphA*) resistance genes, as well as copper (*copE*2*ABDRSE1*), tellurium (*terY3Y2XY1W*, *terZABCDEF*), mercury (*merEDACPTR*) and silver (*silESRCBAP*) resistance gene operons. Likewise, the pK245 plasmid of *S*. Muenster strains carry a class-1 integron (1014 bp), which harbours both aminoglycoside (*aacC2*) and sulphonamide (*dfrA14*) resistance gene cassettes. Furthermore, the pRSF1010-SL1344 (IncQ1) plasmid, predicted in the *S*. London strain, encodes aminoglycoside (*strB*, *aph(3”)-lb*) and sulphonamide (*sul2*) resistance genes; while R27 (IncHI1A), predicted in one *S*. Give strain, encodes resistance factors against tetracycline (*tetA*), magnesium and cobalt *(corA*), as well as ultraviolet light (*mucAB*). Interestingly, although the pNDM-KN (IncA/C2) and pK245 (IncR) plasmids encode class-1 integrons and resistance genes against multiple drugs (i.e. extended spectrum beta lactams, sulphonamides, chloramphenicol and macrolides), these genes were not found in the newly sequenced isolates carrying replicons of these two plasmids.

Predicted plasmids also divided *S*. Montevideo isolates into two groups, one that carried pOLA52 (n = 18) and the other IncFII(p96A) (n = 11). Most of the pOLA52-carrying isolates (n = 14) also had the small pIGJC156 plasmid, which only carries three plasmid replication-related genes. Interestingly, both IncFII(p96A) and pOLA52-carrying isolates had genes encoding a type IVA (VirB/D4) T4SS, which is known to fulfil the translocation of both DNA and effector molecules and is associated with persistent infections in numerous pathogens^23^. This feature was not present in strains of other serovars from the slaughterhouse. Considering *S*. Montevideo was overrepresented in this location, and the fact that the T4SS was putatively carried in plasmids, we reasoned T4SS might be associated with fitness advantages in that niche.

To test this hypothesis, we conducted a sequence analysis among a selected group of 86 additional *S*. Montevideo strains publicly available and originally isolated in Mexico, USA and Canada from a variety of foods, clinical cases, and the environment (accession numbers provided in Table S3). For that purpose, we used the amino acid sequences of each of the T4SS components of one of our strains (SRR3479678) as sequence queries. Strikingly, T4SS components are uncommonly found in only 2/86 isolates: one from an unknown source (WGS project accession AFCS01) and one isolated from ground beef (WGS project accession LHGG01). This observation suggests a variable and possibly niche-related distribution of T4SS in *S*. Montevideo. Moreover, the co-occurrence of T4SS and toxin/antitoxin systems (i. e. *ccdAB*, *stbED*) observed in pOLA52 and IncFII(p96A) plasmids likely ensures all the progeny inherits the plasmid, stabilizing its fitness. In addition, the pOLA52 plasmid carries resistance genes against betalactams (*bla*-TEM) and quinolones (*oqxAB*), which could further contribute to the improved fitness of strains if subjected to selective pressure. Overall, these results emphasise the need of further research to gain insights into the actual contribution of VirB/D4 plasmids to fitness advantages in feedlot cattle.

## Discussion

In this study, we conducted comparative genomics of 59 NTS isolates of 10 different serovars collected along the beef production chain across nine months in different regions of Mexico. Interestingly, *S*. Typhimurium, which is commonly found in beef-associated isolates worldwide^24^, was not detected here. Nevertheless, the distribution of NTS serovars is often irregular across time, studies and regions. For instance, *S. enterica* subsp. *enterica* ser. Meleagridis, Anatum, Agona, and Typhimurium, were among the top 10 most common NTS serovars isolated from retail beef in four states of Mexico in 2002–2005^25^. More recently, some of the serovars we found (i.e. Montevideo, Muenster, Give, and Reading) but not Typhimurium have been reported in bovine-associated samples^26,27^. It is worth noting, however, that the overrepresentation of *S*. Montevideo we observed is consistent with the increasing prevalence of this serovar among healthy cattle in North America^28,29^. Moreover, our *S*. Montevideo strains were isolated from feedlot cattle, which is managed similarly across Canada, Mexico, and the United States. Interestingly, all NTS serovars studied here have been implicated in human clinical cases in Mexico, according to a historical paper of epidemiological data (1972–1999)^30^. Therefore, the significance of non-clinical isolates as reservoirs for human infections should not be minimized.

The SNP-based phylogeny shows NTS disseminates up to retail level and across distant geographical regions and ecological niches within Mexico. These findings highlight the significance of beef cattle as a reservoir of NTS, especially when the pathogen successfully establishes itself in apparently healthy animals that are approved for slaughter. Recent studies have demonstrated that the physiological stress associated with pre-slaughter handling and transport of livestock increases *Salmonella* faecal shedding^31,32^. Moreover, Ginocchio *et al*.^33^ suggested intensive animal production could create a high probability of a continual faecal-oral infection cycle, whereby the pathogen may not require to enter cells of the intestinal epithelium for its successful replication. In this instance, key metabolic activities that are essential for acute salmonellosis might be dispensable during persistent infections, as previously suggested^34^. Likewise, studies in a mouse model showed less invasive NTS strains are excreted at higher concentrations in the faeces^35^. This could be a key driver governing the relative representation of NTS serovars in certain geographical regions. Such hypothesis is supported by a recent study^36^ that suggests the emergence of a cattle-associated subtype of *S. enterica* subsp. *enterica* ser. Cerro in the United States to be associated with subclinical infections and thus, the lack of control measures to contain its spread.

Regarding the virulence genomic profile, most isolates seem to have a conserved virulence machinery. In general, however, isolates that were genotypically closer to *S*. Typhimurium, such as those of *S*. Newport, are the only ones harbouring genes associated with more virulent phenotypes (i. e. *lpfABCDE, sodCI*)^13,14^. This is consistent with the phylogenetic analysis, showing strains of *S*. Newport are closely related to those of *S*. Typhimurium and *S*. Newport implicated in human infections in Mexico.

*Salmonella* pathogenicity islands are essential for virulence expression across livestock species^37^. The different versions of SPIs 1 and 3 observed in some isolates are consistent with previous research documenting both SPIs remain unstable in some *Salmonella* environmental strains. For instance, different studies have reported absence of some genes (i.e. *hilA*)^8^, as well as large deletions of entire loci (i. e. *inv*, *hil*, *spa*)^33^ in SPI-1 of various *S. enterica* subsp. *enterica* serovars. This is in line with the massive deletion of SPI-1 genes we observed in *S*. Reading isolates. Likewise, the deletion of the T3SS effector *avrA* has been frequently reported in less virulent strains^35,38^. Along the same lines, the proteins encoded in the 5′ region of SPI-3 have not been linked to virulent phenotypes and thus, these genes are thought to be subjected to negative selective pressure^39^, leading to their eventual deletion. Moreover, it has been suggested that the instability of this region could be associated with its proximity to the insertion site of SPI-3 (tRNA-*selC*), which is likely a hot spot for foreign DNA integration^37^.

It is difficult to pinpoint if the observed differences in SPIs would result in attenuated virulent phenotypes in bovines. As shown here, despite lacking part of the T3SS apparatus, *S*. Reading isolates were still present in cattle faeces. SPI-4, which was 100% conserved in all isolates, has been shown to play a major role in bovine intestinal colonization^40^. Conversely, it has been demonstrated that disruption of several genes of SPIs 1, 2 and 3 do not affect colonization of chicken, pigs, and cattle, probably due to the functional redundancy in several *Salmonella* metabolic pathways^5^. In this regard, for instance, both *avrA* and *sopB* inhibit cell death during the first phase of the infection^17^. Likewise, since iron acquisition is essential for survival and pathogenesis of most bacteria, they usually carry multiple iron acquisition and metabolism genes. As observed here, strains of *S*. Reading have a truncated *sitD* gene, which encodes an iron transporter. This modification has not been reported before, to the best of our knowledge. However, these isolates carry a highly conserved repertoire of other iron acquisition and metabolism genes (*iroBCDE, fepBCDEG, fhuBCD, exbBD, and tonB*) that may compensate the lack of a functional SitD.

The typhoidal toxin genes that were widely distributed in our isolates have been reported previously in few NTS serovars^21,41^. Among them, the *cdtB* islet has received most attention since its functionality in NTS could improve our understanding of typhoid fever pathogenesis, where the typhoid toxin is thought to play a central role^42^. However, literature findings regarding the function of the typhoid toxin in NTS are not conclusive. On one hand, several *in vitro* studies have demonstrated the cytotoxicity phenotype of NTS isolates carrying the *cdtB* islet^43,44^. Therefore, these authors conclude CdtB produced by NTS appears to play an important role in pathogenesis. On the other hand, the typhoid toxin has been shown to favour host survival and long-term infections in a mouse model^45^. Likewise, research showed *cdtB*-positive NTS strains of *S*. Montevideo and *S*. Schwazergrund, accounted for invasive disease in humans^46^, showed lower invasiveness and did not cause mortality in intraperitoneally infected mice as compared to *cdtB*-negative virulent strains (i.e. *S. enterica* ser. Dublin, Cholerasuis, Typhimurium, and Enteritidis).

Although phylogenetic analysis did not support these toxin genes are source-associated, their widespread distribution in non-clinical isolates of bovine origin is intriguing. Possibly, these genes are not expressed in NTS adapted to an extracellular lifestyle, as commented before^34^. However, their high conservation across NTS serovars of multiple sources indicates they should be relevant for pathogen survival and/or for the interaction with host cells. Otherwise, these horizontally-acquired regions would have been progressively degraded or deleted from the genome. Undoubtedly, further research is needed to further understand the function of typhoidal toxins in NTS, as well as to test if they provide fitness advantages in certain hosts or livestock production settings.

Plasmids, another factor that contribute to environmental fitness in bacteria^47^, are particularly prone to have a bearing at the population level. Interestingly, the strains with a wider geographical distribution (*S*. Give and *S*. Muenster) were predicted to carry resistance plasmids that contributed antimicrobial and heavy metal resistance genes. This could also represent fitness advantages under intensive beef production, where the use of high rates of mineral supplementation is commonly practiced^48^. For instance, recent research has shown the occurrence of a transferable copper resistance gene (*tcrB*) in faecal enterococci is higher (~10x) in feedlot cattle fed copper-supplemented diets (100 mg/kg of feed) as compared to those fed normal copper levels (10 mg/kg of feed)^49^. Likewise, Zhou et al^50^. observed the use of copper and zinc supplementation in the diet of dairy cattle was related with the abundance of metal resistance genes (r = 0.69, p < 0.01) and antibiotic resistance genes (r = 0.62, p < 0.01) in gut bacteria. This evidence highlights the importance of avoiding excessive livestock mineral supplementation, which may indirectly contribute to pathogen persistence in the host and/or the environment.

Along the same lines, it is interesting to note that the presence of T4SS is associated with persistent infections in numerous pathogens^23^. Based on this observation, we speculated that the presence of T4SS, exclusively in *S*. Montevideo isolates, might have contributed to the overrepresentation of this serovar among slaughterhouse samples. This hypothesis is supported by recent *in vitro* studies showing strains of *S. enterica* subsp. *enterica* ser. Heidelberg carrying a plasmid-borne T4SS down regulate host innate immune response, which allows a higher invasion rate as compared to T3SS-mediated invasion^51^. Conversely, studies with strains of the same serovar in a *Caenorhabditis elegans* model^52^, showed T4SS were associated with a greater pathogenic potential. To the best of our knowledge, there are no previous reports of VirB/D4 plasmids in *S*. Montevideo.

Overall, this study provides evidence of clonal dissemination and persistence of NTS, originated from apparently healthy animals, along the food production chain. Most of these strains carry a conserved repertoire of virulence factors, which poses a food safety risk to consumers. Moreover, comparative genomic analyses show non-clinical NTS isolates may act as reservoirs of genetic variability in the environment, influencing the organism’s characteristics as a foodborne pathogen. Further research is needed for a better understanding of the evolution and survival of bovine-associated *Salmonella*, especially at the pre-harvest phase, with emphasis in the features that are widely distributed in the population and may contribute to host adaptation and persistence.

## Methods

### Bacterial strains

We used a panel of 59 NTS isolates collected during previous studies, which were part of two different master’s theses, conducted in 2013 by our research group (Fig. 1). In one of the studies, we isolated 39 NTS strains from beef cattle rectal contents (34/100), carcass swabs (3/100), and primal cut swabs (2/100) from a commercial beef slaughter operation located in northern Mexico^53^. We also included a group of unrelated strains, isolated from retail ground beef (20/150) during the second master’s thesis project^54^. These isolates were collected from retailers located in Mexico City and Guadalajara, more than 2,000 km away from the northern slaughterhouse. Hence, our study included isolates collected along the beef production continuum and from distant geographical locations, providing the basis for studying their genetic diversity, as well as the potential role of genetic determinants in the relative representation and dissemination of specific strains.

All strains were isolated from different carcasses, cuts or ground meat packages across a 9-month period (Table 2). They were also confirmed by conventional biochemical tests (triple sugar iron, sulfhydric acid-indole motility, Simmons citrate, urease, methyl red/Vosges-Proskauer, malonate-phenlylalanine, and gluconate) and polymerase chain reaction targeting the *invA* gene^55^.

**Table 2:** Total number of isolates collected by *Salmonella* serovar, and number of isolates by isolation source, geographical location, and collection date.

*Salmonella* serovar^a^	n	Isolation source	Geographical location	Collection date
Montevideo	29			
	1	Carcass	Mexicali	09/20/2013
	1	Carcass	Mexicali	09/23/2013
	1	Carcass	Mexicali	09/25/2013
	1	Faeces	Mexicali	09/21/2013
	14	Faeces	Mexicali	09/27/2013
	1	Carcass	Mexicali	09/27/2013
	10	Faeces	Mexicali	09/21/2013
Give	8			
	4	Ground beef	Mexico City	04/28/2013
	4	Ground beef	Guadalajara	09/16/2013
Muenster	6			
	1	Primal cut	Mexicali	09/25/2013
	2	Faeces	Mexicali	09/27/2013
	2	Ground beef	Mexico City	03/04/2013
	1	Ground beef	Mexico City	11/04/2013
Newport	4			
	2	Faeces	Mexicali	09/21/2013
	2	Faeces	Mexicali	09/27/2013
Derby	4			
	1	Ground beef	Mexico City	04/28/2013
	2	Ground beef	Mexico City	03/04/2013
	1	Ground beef	Mexico City	04/18/2013
Reading	2	Faeces	Mexicali	09/27/2013
Roodepoort	*2*	Ground beef	Guadalajara	09/16/2013
Senftenberg	2			
	1	Ground beef	Mexico City	11/04/2013
	1	Ground beef	Mexico City	14/18/2013
Bergen	1	Faeces	Mexicali	09/27/2013
London	1	Ground beef	Guadalajara	09/16/2013

^a^Predicted with SeqSero software^12^.

Pure isolates were preserved long term (about 1 year) at −80 °C in vials containing a 50% glycerol solution. Bacteria were recovered from the glycerol stock by streaking onto tubes containing semi-solid tripticase soy agar. Subsequently, the tubes were shipped to the molecular biology laboratory of the Center for Food Safety and Applied Nutrition (Food and Drug Administration, Maryland, USA) for WGS. The input material for WGS was a cell pellet of a 1 mL bacterial culture grown in Luria Bertani broth at 37 °C overnight.

### Whole genome sequencing and serovar prediction

Genomic DNA was extracted with the fully automated Qiagen QIAsymphony system using the QIAsymphony DSP DNA Kit. Next, we quantitated the extracted DNA by Qubit Fluorometric Quantitation (LifeTechnologies), per the manufacturer’s instructions. Finally, DNA libraries were prepared from 1 ng of genomic DNA using the Nextera XT DNA Sample Preparation Kit v.2 (Illumina) and sequenced on the Illumina MiSeq system (paired-end 2 × 250 bp reads). Raw sequences were deposited at the NCBI Sequence Read Archive (SRA) web site and are also available at the Enterobase web server.

For serovar prediction, we conducted *in silico* analysis using raw reads with the SeqSero software^12^. There were isolates where the predicted antigenic profile was incomplete. In these cases, the serovar was estimated by checking that of the closest organisms in the *Salmonella* SNP tree from the NCBI pathogen detection isolates browser (https://www.ncbi.nlm.nih.gov/pathogens/isolates#/search). The accession numbers, strains’ metadata, and the predicted serovars and antigenic profiles are listed in Supplementary Table S4.

Before genome assembly, the quality of raw reads was assessed with the FastQC software^56^. Next, we used Trimmomatic^57^ for removing Illumina adaptors and filter reads according to quality criteria. The trimmed sequences were then re-run in the FastQC software to make sure only high-quality sequences (i.e. quality scores ≥30) were used for genome assembly.

### Genome assembly and annotation

Trimmed sequences were *de novo* assembled in the PATRIC web server^58^ using the SPAdes assembly algorithm^59^. Genome annotation was performed in the RAST server in September 2016^60,61^. The analysis was set up to correct automatically errors and frameshifts. The assembled genomes had 28–109 contigs, an average depth of coverage that ranged from 24 to 268x (median 94x) across strains, while the median N50 and L50 values were 390,205 bp and 5, respectively. Moreover, genome annotation showed G + C content (around 52%) and gene density (about 1000 genes/Mb) were comparable across strains. The full report of assembly and annotation statistics is provided as Supplementary Information (Table S1).

### Genetic relatedness among the newly sequenced NTS

The genetic diversity of NTS strains was assessed through SNP phylogeny. First, SNPs were called, filtered, and validated through CSI Phylogeny 1.4^62^. The resulting concatenated alignment was then used to generate a ML tree in RAxML 7.7.1^63^ under the GTR+ Γ model of nucleotide evolution at the CIPRES web server^64^. The best tree was estimated by RAxML rapid bootstrapping (100 iterations) and subsequent ML search. The resulting tree was visualized in FigTree 1.4.3, and edited with Inkscape 0.91. The same methodology was used for the phylogenetic analysis that included 47 additional isolates from different sources within Mexico. These isolates are publicly available and were collected from produce, pet food, seafood, the environment, and clinical cases (Fig. 2). Two isolates of *S. enterica* subsp. *enterica* serovar Dublin, which is highly adapted to bovines, were also included in the analysis. We did not include isolates of *S*. Bergen and *S*. Roodepoort since no strains of these two serovars were available for comparison.

### Virulence and stress response genomic profile of sequenced isolates

Annotated genomes were screened for the presence of major *Salmonella* virulence factors (adherence, antivirulence, magnesium uptake, regulation, resistance to antimicrobial peptides, serum resistance, stress protein, toxins, and macrophage-inducible gene-5), as reported in the virulence factors data base^65^.

Stress response genes were also analysed, using *Salmonella* Typhimurium LT2 as a reference. This included genes related to heat shock (*rpoH*), acid tolerance response (*rpoS*, *adA*), desiccation stress (*proP*), and fatty acid-associated osmo-tolerance (*fabAB*). The amino acid sequence of each reference protein was BLAST-searched against the annotated genome of each strain, with a maximum e-value threshold of 10^−30^ at the RAST web server^66^. Matching protein sequences were mapped back to their corresponding genes in The Seed Viewer. The resulting amino acid identity percentage was used to build a heatmap, showing the virulence and phylogenetic profiles of the strains, with the aid of MORPHEUS software (https://software.broadinstitute.org/morpheus).

For ambiguous annotations and amino acid identities below 90%, a Psi-Blast analysis was performed from within The Seed Viewer, to corroborate the matching protein was a homologue of the reference protein. Furthermore, when missing proteins were found, the absence of the corresponding genes was verified by comparing reference genes against the raw reads of the involved isolate with the Artemis Comparison Tool^67^. Variation within SPIs was also assessed. For that purpose, we collected the nucleotide and protein sequences of SPIs 1 through 5 of *Salmonella* Typhimurium LT2 from the Pathogenicity Island Database^16^. Next, we prepared a multi-FASTA file with the reference sequences, which was used in the BLAST Ring Image Generator Software, version 0.95^68^, against the assembled genomes to produce the corresponding BLAST atlas. We used the LT2 strain as a reference for it has been widely characterized and its whole genome is available at NCBI. For those SPIs showing variation across strains, their reference protein sequences were compared to those of our genomes at the GView web server^69^, with the following configuration: expect e-value cutoff = 0.001, genetic code = bacterial and plant plastid, alignment length cutoff = 50, percent identity cutoff = 70 and tblastx as the BLAST program. Moreover, we conducted a conserved domain (CD) search (https://www.ncbi.nlm.nih.gov/Structure/cdd/wrpsb.cgi) to analyse if the truncated SitD of *S. enterica* ser. Reading isolates still matched an ABC-type Mn^2+^/Zn^2+^ transport system component. Since it did, we then analysed the reference protein (H9L416) structure at Uniprot (http://www.uniprot.org/) to check if the untranslated fragment contains regions that are required for a fully functional protein.

Finally, since the virulence profile showed a widespread dissemination of SPIs 11 and 18 among strains of the major sublineage, we also used the concatenated amino acid sequences of representative genes from both SPIs for phylogenetic reconstruction, including 63 additional isolates publicly available at NCBI (Fig. 5). The criteria used for the inclusion of isolates in this analysis were: (1) strains that are positive for both SPI-11 and SPI-18, (2) either complete genomes or draft genomes with full genome representation in its final version, (3) depth of coverage of at least 30x, and 4) genes from both SPIs should be annotated in the genome. Since *pltB* and *taiA* were not annotated in many isolates, we decided to conduct the phylogenetic analysis with the two biggest proteins of SPI-11 (CdtB and PltA), and HlyE from SPI-18. We also included representative virulent strains (Typhi CT18, Paratyphi A, and Typhimurium sal199), as well as three strains of *S. enterica* subsp. *diarizonae* (CP011288.1, CP023345.1, and MLXQ00000000.1).

The concatenated sequences of CdtB, PltA, and HlyE were aligned in Seaview^70^ through ClustalO^71^ and curated with Gblocks^72^. The resulting Nexus file was used to construct a phylogenetic tree using MrBayes 3.2.6^73^ with the following parameters: aamodelpr = mixed, samplefreq = 100, burninfrac = 0.25 in four chains and for 1 million generations. The output tree was edited with InkScape 0.91 software (https://inkscape.org/es/).

### *In silico* plasmid prediction

First, plasmids were predicted with the aid of PlasmidFinder 1.3^74^. The analysis was carried out with the raw reads at the Center for Genomic Epidemiology web server using a threshold identity of 95%. When plasmid replicons were detected, these were considered candidate plasmids. Accordingly, we used the accession numbers of the prediction output to collect their complete nucleotide sequence from NCBI for further confirmation. Afterwards, we aligned the nucleotide sequence of each reference plasmid against the assembled genomes at the GView web server^69^. The candidate plasmids were confirmed based on the identification of consecutive genes that were homologous between contigs and plasmids. If most of the candidate plasmid (≥70%) was covered in the assembled genome, it was considered as a putative plasmid. Finally, we used the average depth of coverage to verify the above prediction. Usually, plasmid-associated contigs have very similar or the same read depth, which is also higher than those of chromosomal contigs (except for multi-copy ribosomal genes). We used this procedure since draft genomes contain both chromosomal and plasmid DNA. However, we acknowledge conducting *in silico* plasmid predictions with these data is cumbersome and not fully reliable. The plasmids may be scattered in multiple contigs or some of their fragments might be lost in the gaps. Additionally, low-copy plasmids may be overlooked due to its similar depth of coverage in relation to chromosomal DNA. Hence, these should be considered putative results until the predicted plasmids are closed.

### Data availability

The data sets analysed during the current study are available in the NCBI repository (https://www.ncbi.nlm.nih.gov/sra). The accession numbers are provided either in Table 2 (for our own isolates) or in the methods section (for additional publicly available genomes that were included in some comparative genomic analyses). Furthermore, our isolates are also available in the Enterobase repository (https://enterobase.warwick.ac.uk/species/index/senterica) with the accession numbers provided in Table 2. Likewise, data generated during this study are either included in this published article (and its Supplementary Information files) or are available from the corresponding author on reasonable request.

## Electronic supplementary material

Supplementary figures

Table S1

Table S2

Table S3

Table S4
