# Supplementary material for: Whole genome sequencing reveals widespread distribution of typhoidal toxin genes and VirB/D4 plasmids in bovine-associated nontyphoidal Salmonella
Source: Sci Rep. 2018 Jun 29;8:9864. doi: 10.1038/s41598-018-28169-4 (PMC6026178; doi:10.1038/s41598-018-28169-4)
Supplement: Supplementary file 1 — Supplementary figures [file 41598_2018_28169_MOESM1_ESM.pdf]

Whole genome sequencing reveals widespread distribution of typhoidal toxin genes and VirB/D4 plasmids in bovine-associated nontyphoidal *Salmonella*

Enrique Jesús Delgado-Suárez[1,*], Nelly Selem-Mojica[2], Rocío Ortíz-López[3], Wondwossen A. Gebreyes[4], Marc W. Allard[5], Francisco Barona-Gómez[2,*], and María Salud Rubio-Lozano[1]

[1]Facultad de Medicina Veterinaria y Zootecnia, Universidad Nacional Autónoma de México, Mexico City, 04510, Mexico
[2]Evolution of Metabolic Diversity Laboratory, Unidad de Genómica Avanzada (Langebio), Cinvestav-IPN, Irapuato, 36821, Mexico
[3]Centro de Investigación y Desarrollo en Ciencias de la Salud, Universidad Autónoma de Nuevo León, Monterrey, 66460, Mexico
[4]College of Veterinary Medicine,The Ohio State University, Columbus, 43210, USA
[5]Office of Regulatory Sicence, Center for Food Safety and Applied Nutrition, U. S. Food and Drug Administration, College Park, 20740, USA

*Corresponding authors:

Enrique Jesús Delgado Suárez

Facultad de Medicina Veterinaria y Zootecnia,

Universidad Nacional Autónoma de México,

Av. Universidad 3000 Col. UNAM, C.U., Coyoacán 04510 Ciudad de México, México

Telephone: (52)(55)5540741954

E-mail: enriquedelgado.suarez@gmail.com

Francisco Barona Gómez

Evolution of Metabolic Diversity Laboratory,

Unidad de Genómica Avanzada (Langebio), Cinvestav-IPN

Km. 9.6 Libramiento Norte Carr. Irapuato-León Irapuato, 36824, Mexico

Telephone: (52)(462)6239600

E-mail: franciso.barona@cinvestav.mx

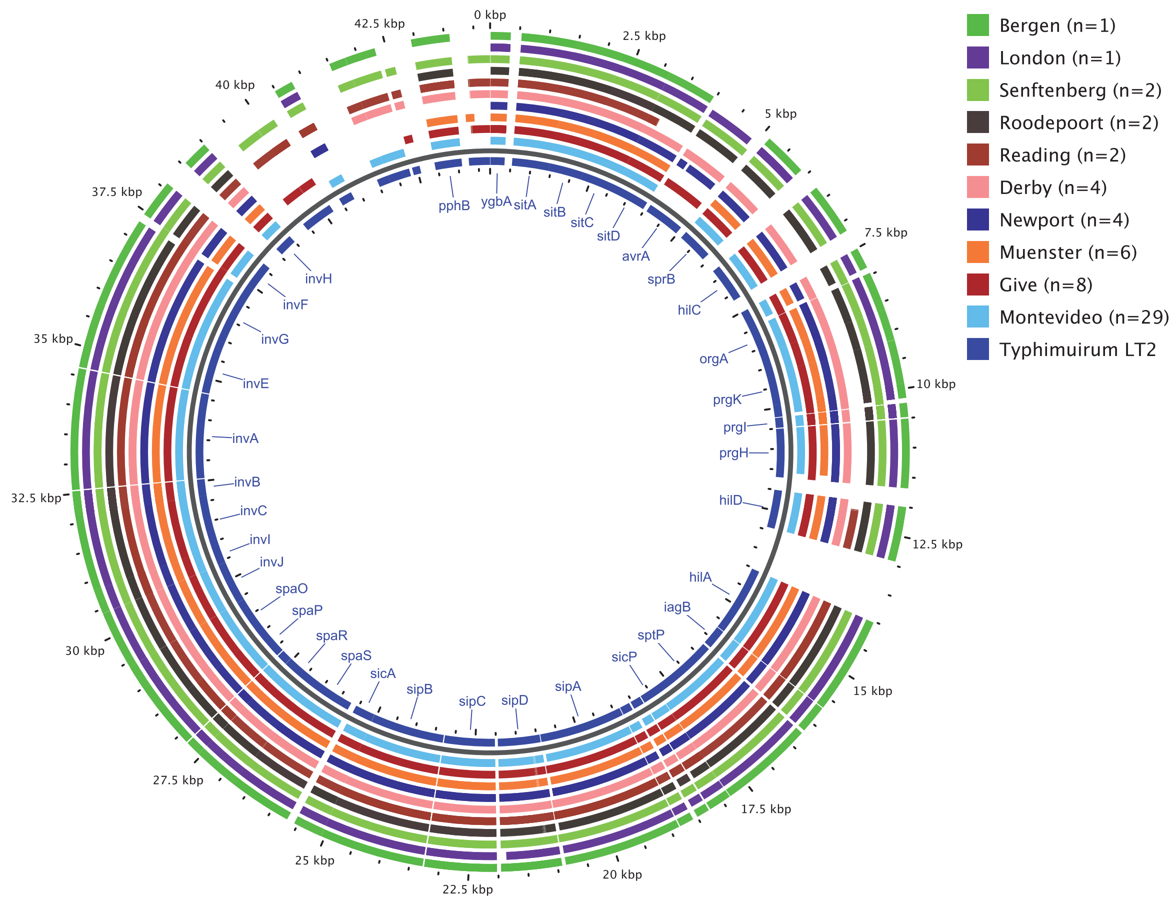

Figure S1. BLAST atlas of SPI-1 encoded proteins of 59 nontyphoidal *Salmonella* strains of different serovars using *Salmonella* Typhimurium LT2 as a reference. The black slot corresponds to the backbone and the inner ring to the reference proteins. Map generated with GView, version 1.7 through the tblastx program, with e-value=0.001, alignment cutoff=50, identity cutoff=70, and no filtering of low complexity sequences.

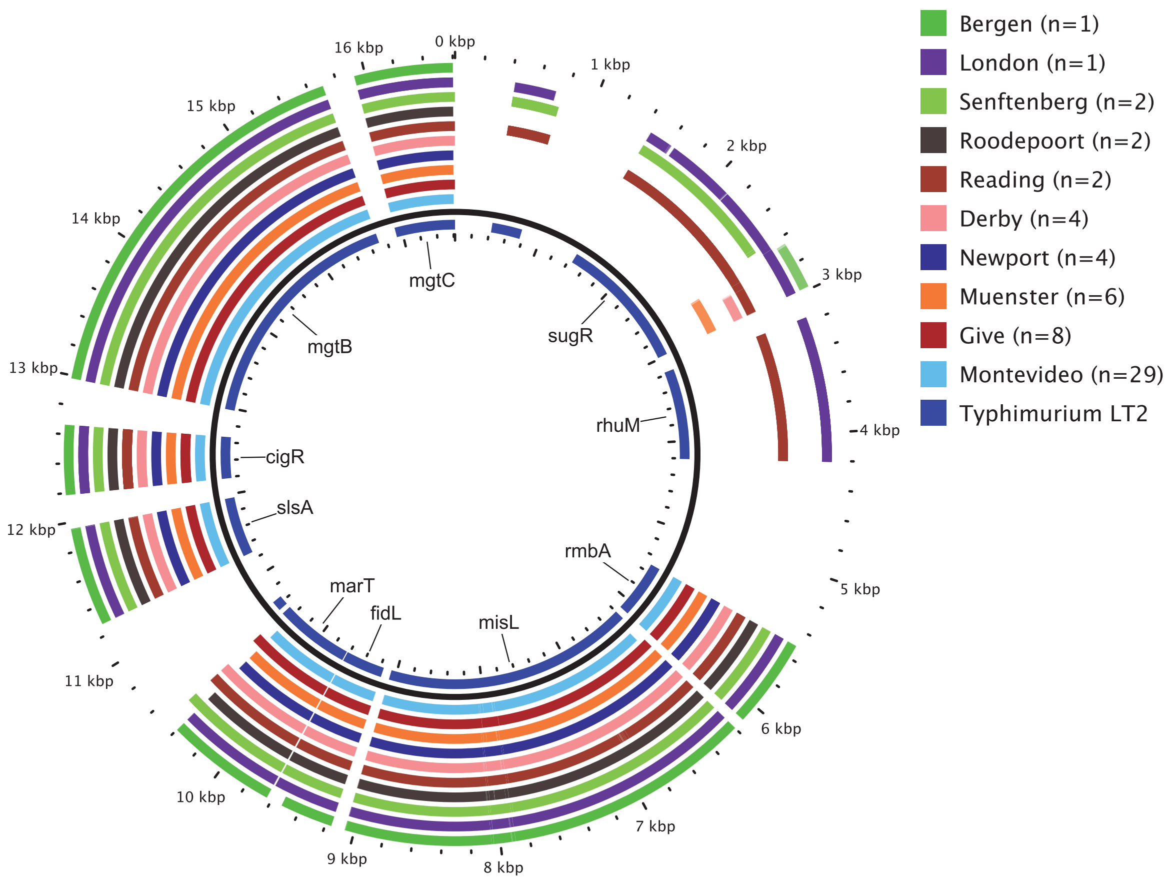

Figure S2. BLAST atlas of SPI-3 encoded proteins of 59 nontyphoidal *Salmonella* strains of different serovars using *Salmonella* Typhimurium LT2 as a reference. The black slot corresponds to the backbone and the inner ring to the reference proteins. Map generated with GView, version 1.7 through the tblastx program, with e-value=0.001, alignment cutoff=50, identity cutoff=70, and no filtering of low complexity sequences.

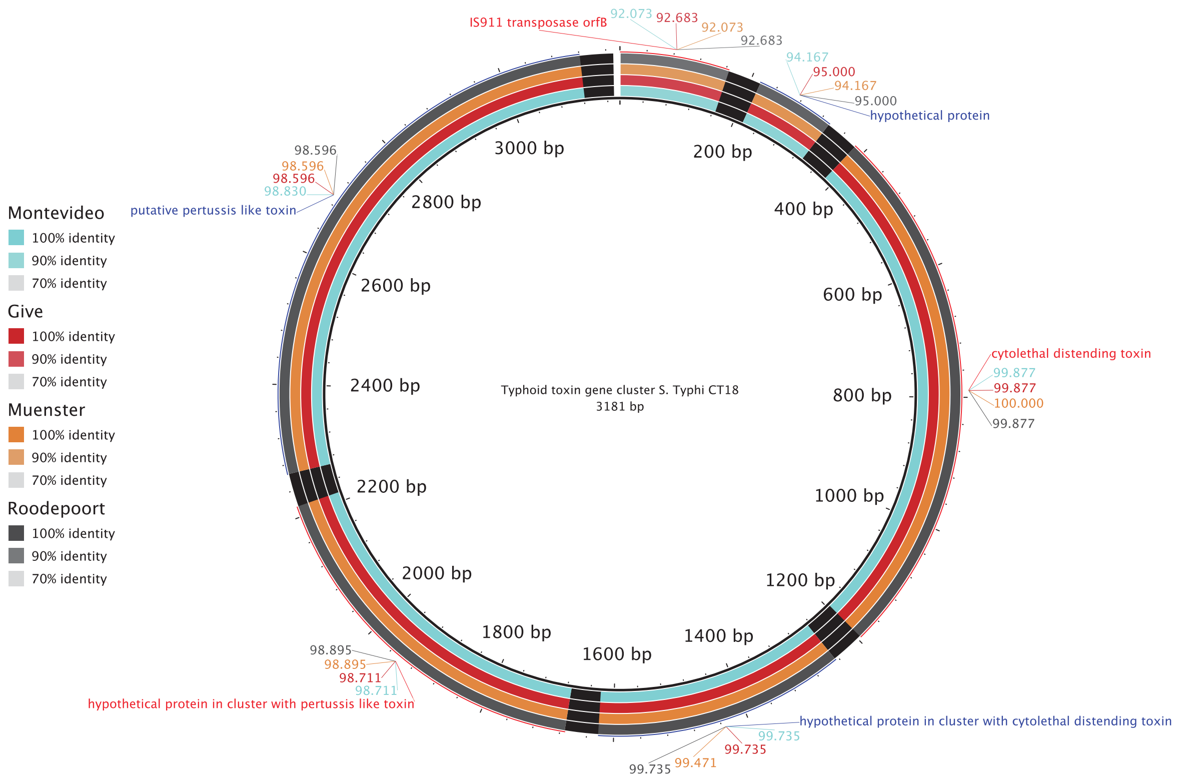

Figure S3. BLAST atlas of SPI-11 encoded proteins of several nontyphoidal *Salmonella* isolates carrying typhoid toxins. *Salmonella* Typhi CT18 was used as a reference. The black slot corresponds to the backbone. The percentage of amino acid identity of each serovar to the reference proteins is indicated according to the color code shown in the legend. Map generated with BRIG version 0.95.

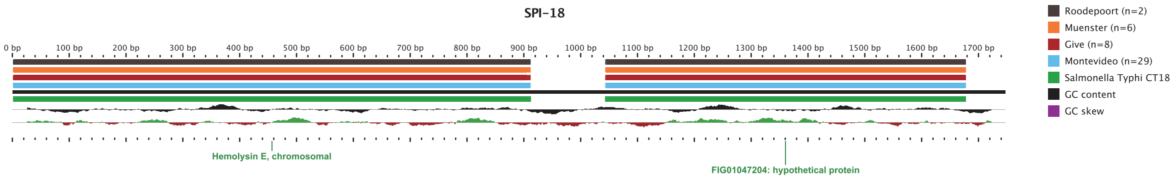

Figure S4. BLAST atlas of SPI-18 encoded proteins of several nontyphoidal *Salmonella* isolates carrying typhoid toxins. *Salmonella* Typhi CT18 was used as a reference. Map generated with GView, version 1.7 through the tblastx program, with e-value=0.001, alignment cutoff=50, identity cutoff=70, and no filtering of low complexity sequences.

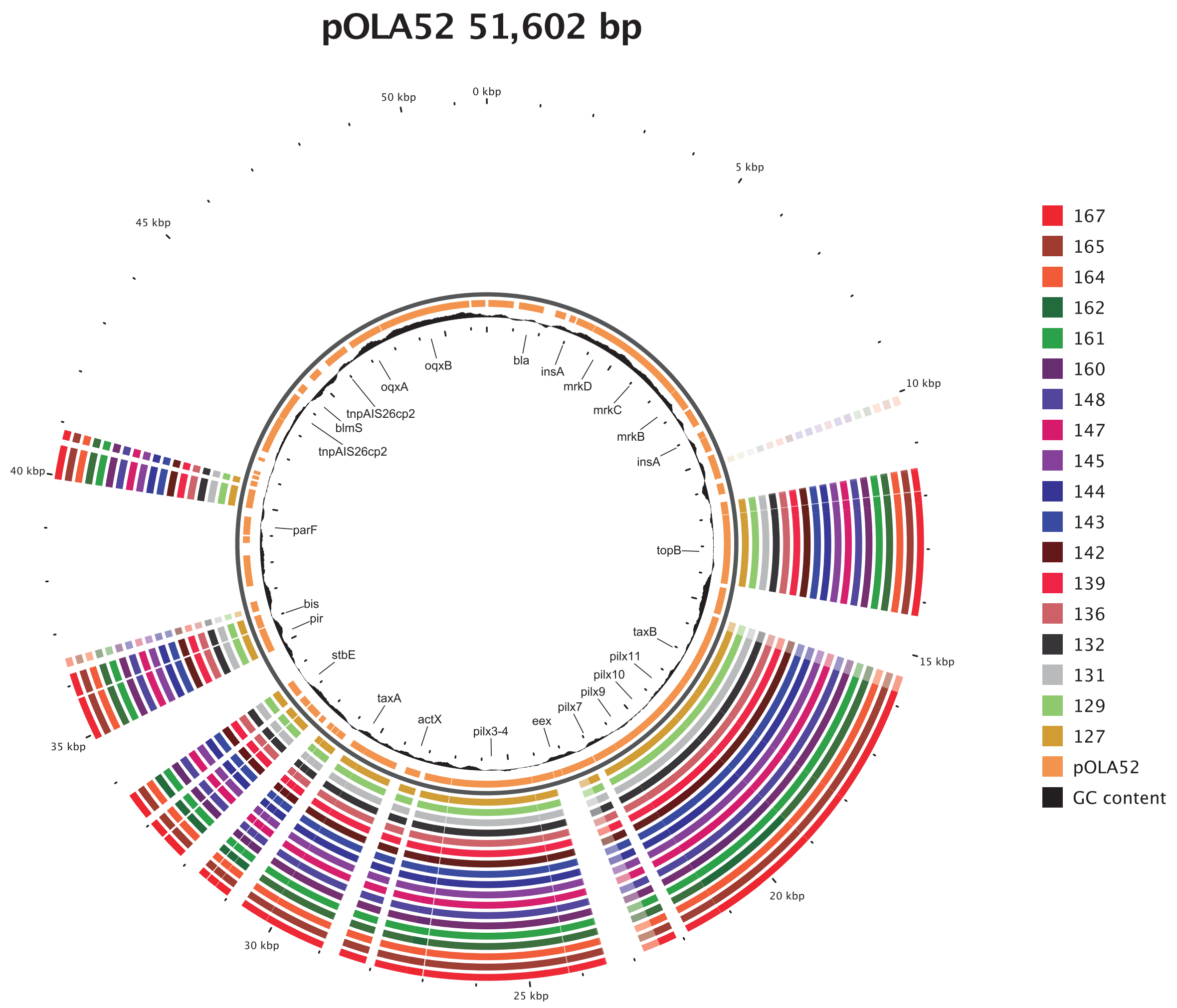

Figure S5. Alignment of reference plasmid pOLA52 to the contigs of the newly sequenced *Salmonella* Montevideo isolates (see Table 2 for accession numbers) where this plasmid was predicted by the PlasmidFinder tool. Map generated with GView, version 1.7 through the tblastx program, with e-value=0.001, alignment cutoff=50, identity cutoff=70, and no filtering of low complexity sequences.

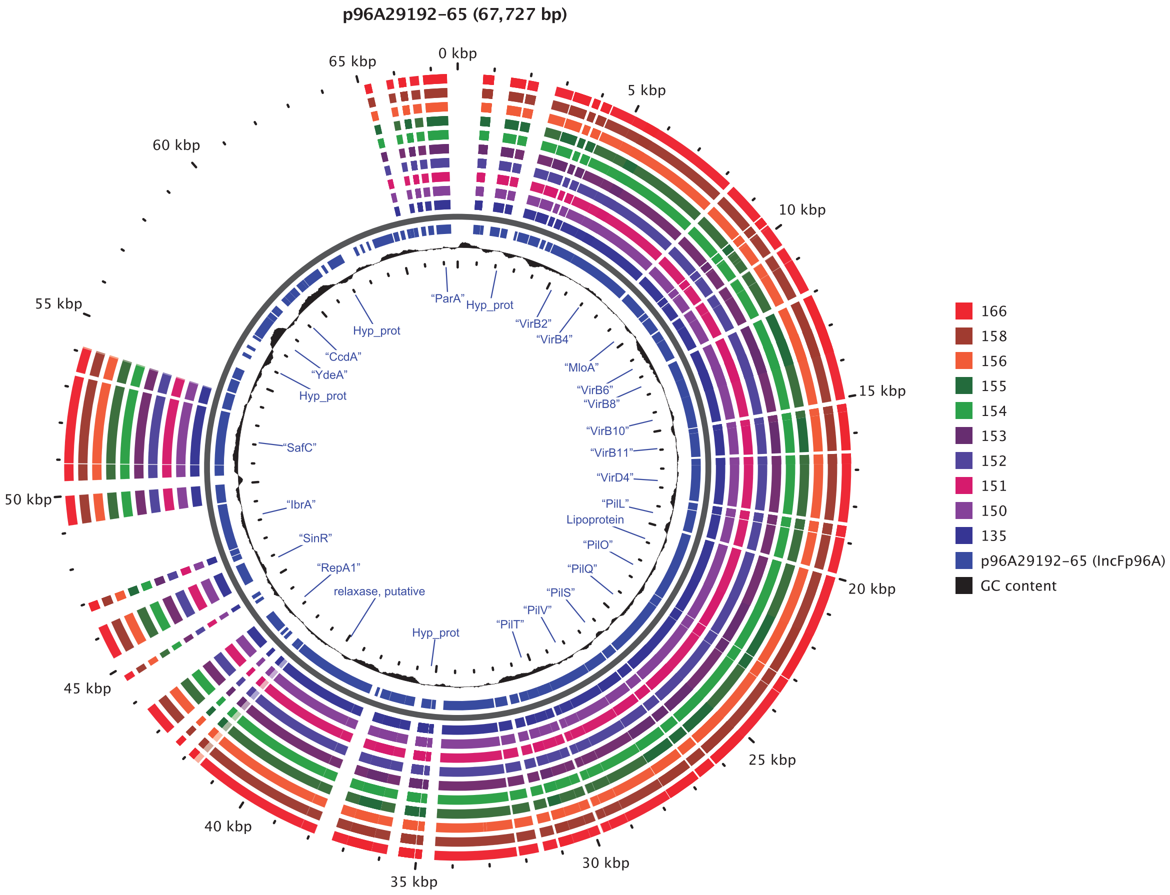

Figure S6. Alignment of reference plasmid p96A29192-65 (IncFp96A) to the contigs of the newly sequenced *Salmonella* Montevideo isolates (see Table 2 for accession numbers) where this plasmid was predicted by the PlasmidFinder tool. Map generated with GView, version 1.7 through the tblastx program, with e-value=0.001, alignment cutoff=50, identity cutoff=70, and no filtering of low complexity sequences.

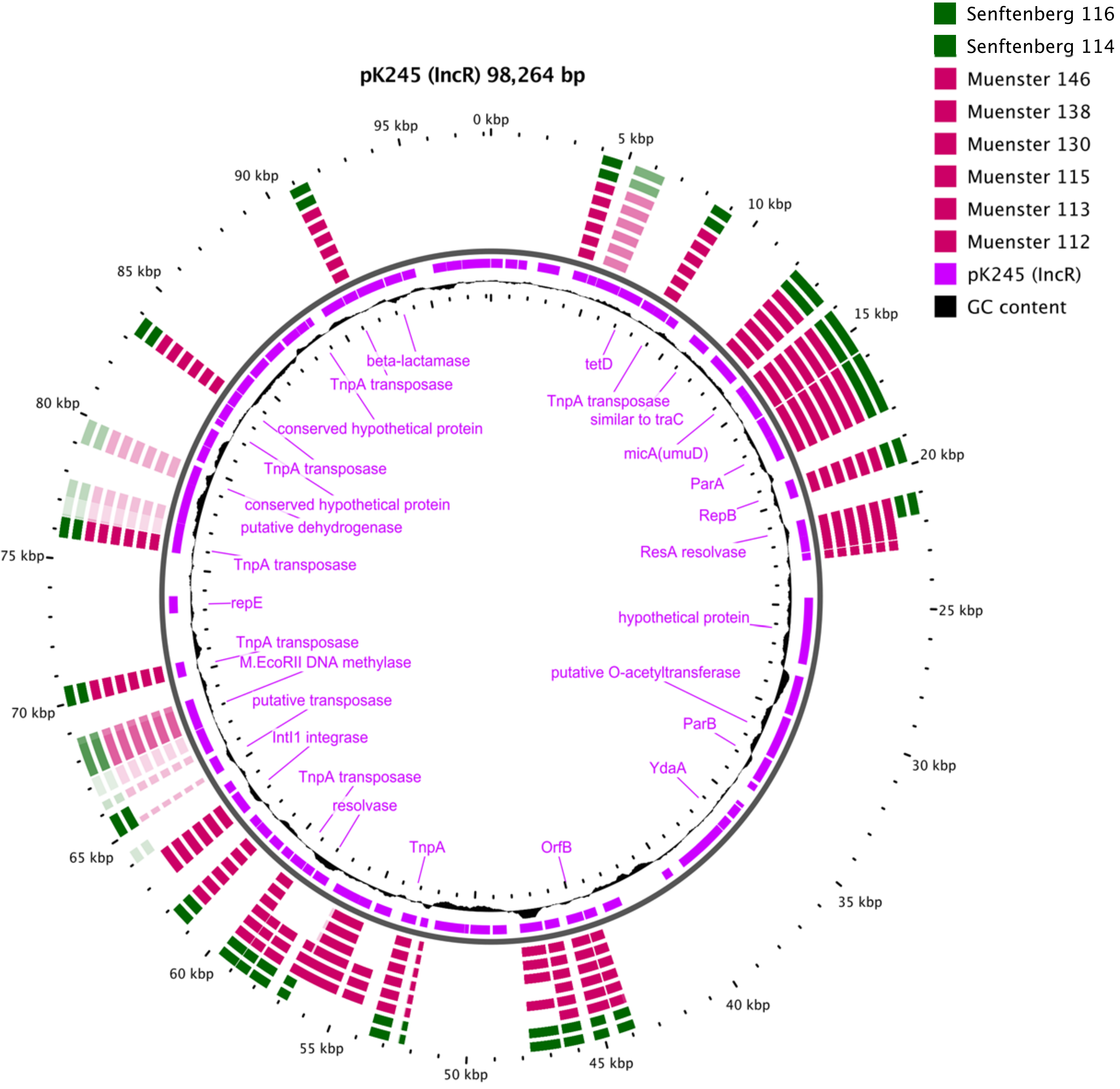

Figure S7. Alignment of reference plasmid pK245 (IncR) to the contigs of the newly sequenced S. enterica ser. Muenster and Senftenberg isolates (see Table S1 for accessions) where this plasmid was predicted by the PlasmidFinder tool. Map generated with GView, version 1.7 through the tblastx program, with e-value=0.001, alignment cutoff=50, identity cutoff=70, and no filtering of low complexity sequences.

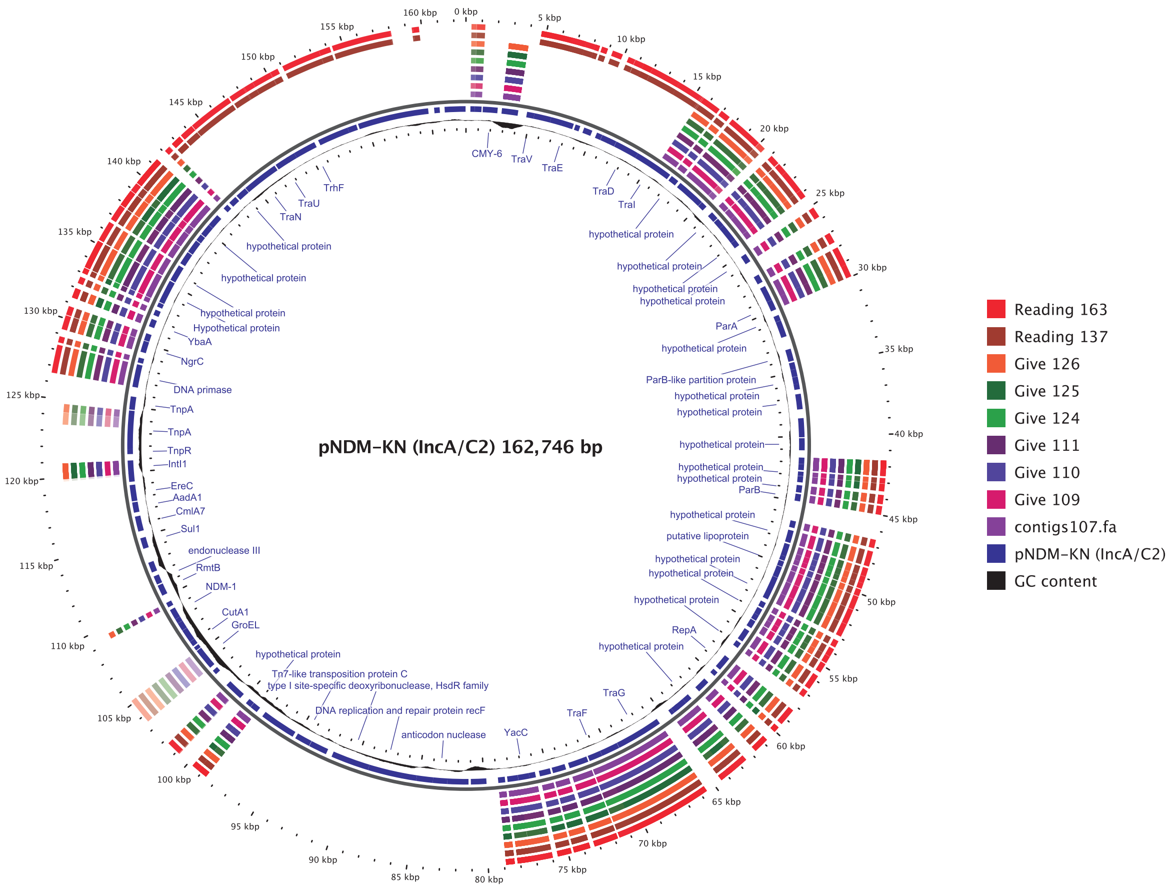

Figure S8. Alignment of reference plasmid pNDM-KN (IncA/C2) to the contigs of the newly sequenced *Salmonella* Give and Reading isolates (see Table 2 for accession numbers) where this plasmid was predicted by the PlasmidFinder tool. Map generated with GView, version 1.7 through the tblastx program, with e-value=0.001, alignment cutoff=50, identity cutoff=70, and no filtering of low complexity sequences.

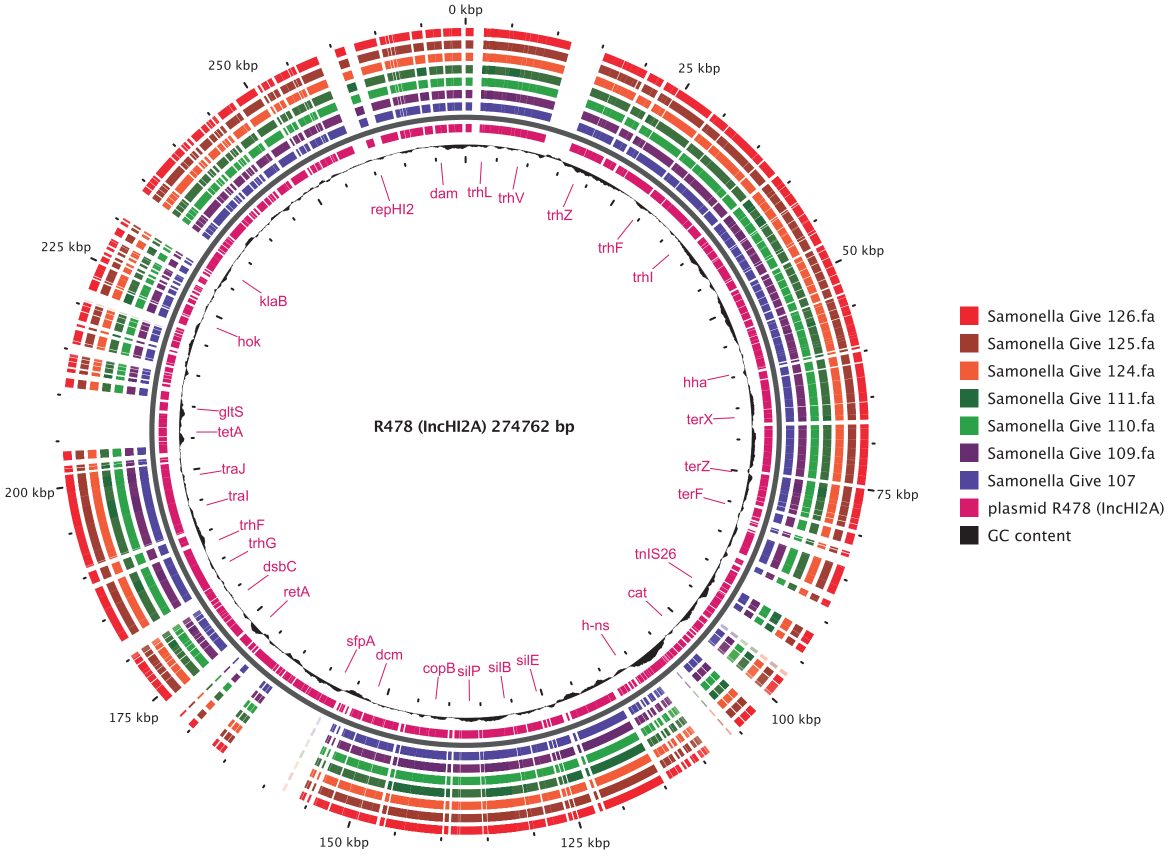

Figure S9. Alignment of reference plasmid R478 (IncHI2A) to the contigs of the newly sequenced *Salmonella* Give isolates (see Table 2 for accession numbers) where this plasmid was predicted by the PlasmidFinder tool. Map generated with GView, version 1.7 through the tblastx program, with e-value=0.001, alignment cutoff=50, identity cutoff=70, and no filtering of low complexity sequences.

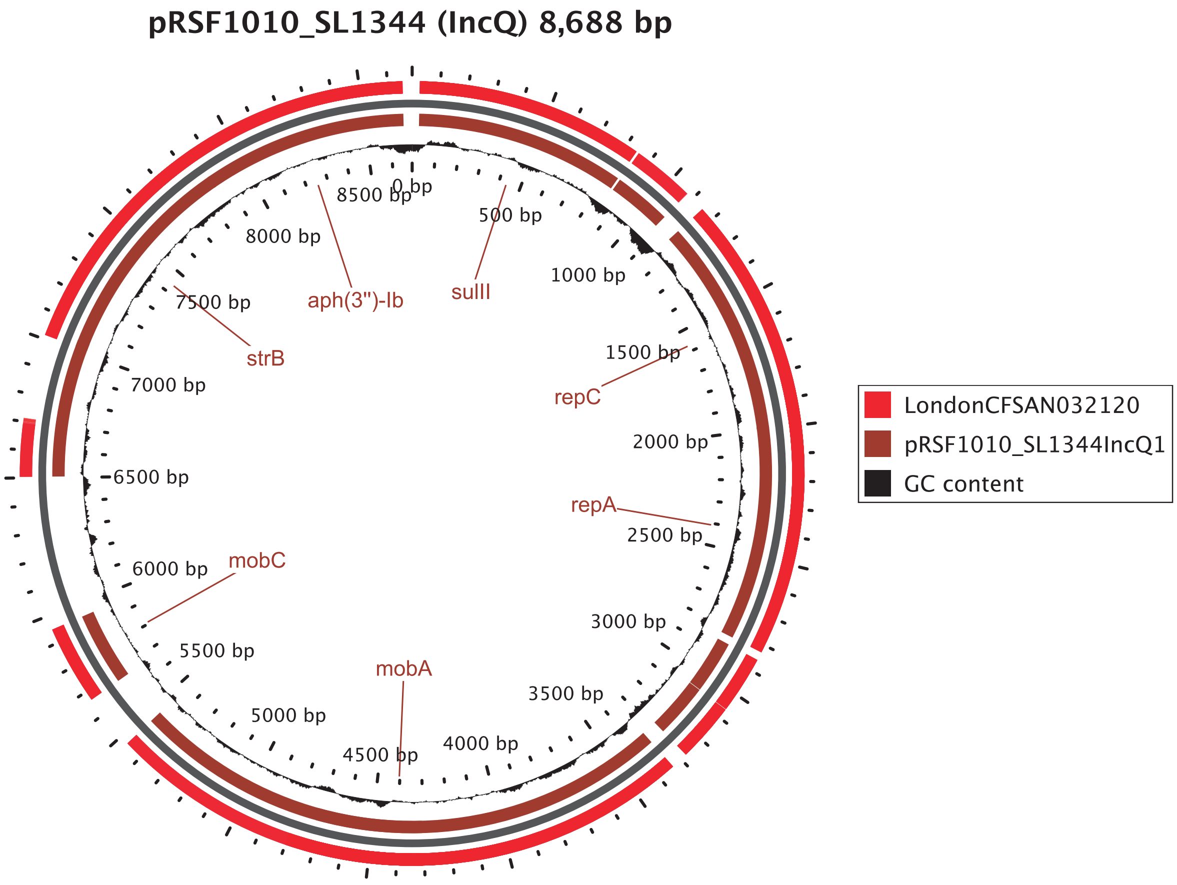

Figure S10. Alignment of reference plasmid pRSF1010_SL1344 (IncQ1) to the contigs of the newly sequenced *Salmonella* London isolate (see Table 2 for accession number) where this plasmid was predicted by the PlasmidFinder tool. Map generated with GView, version 1.7 through the tblastx program, with e-value=0.001, alignment cutoff=50, identity cutoff=70, and no filtering of low complexity sequences.

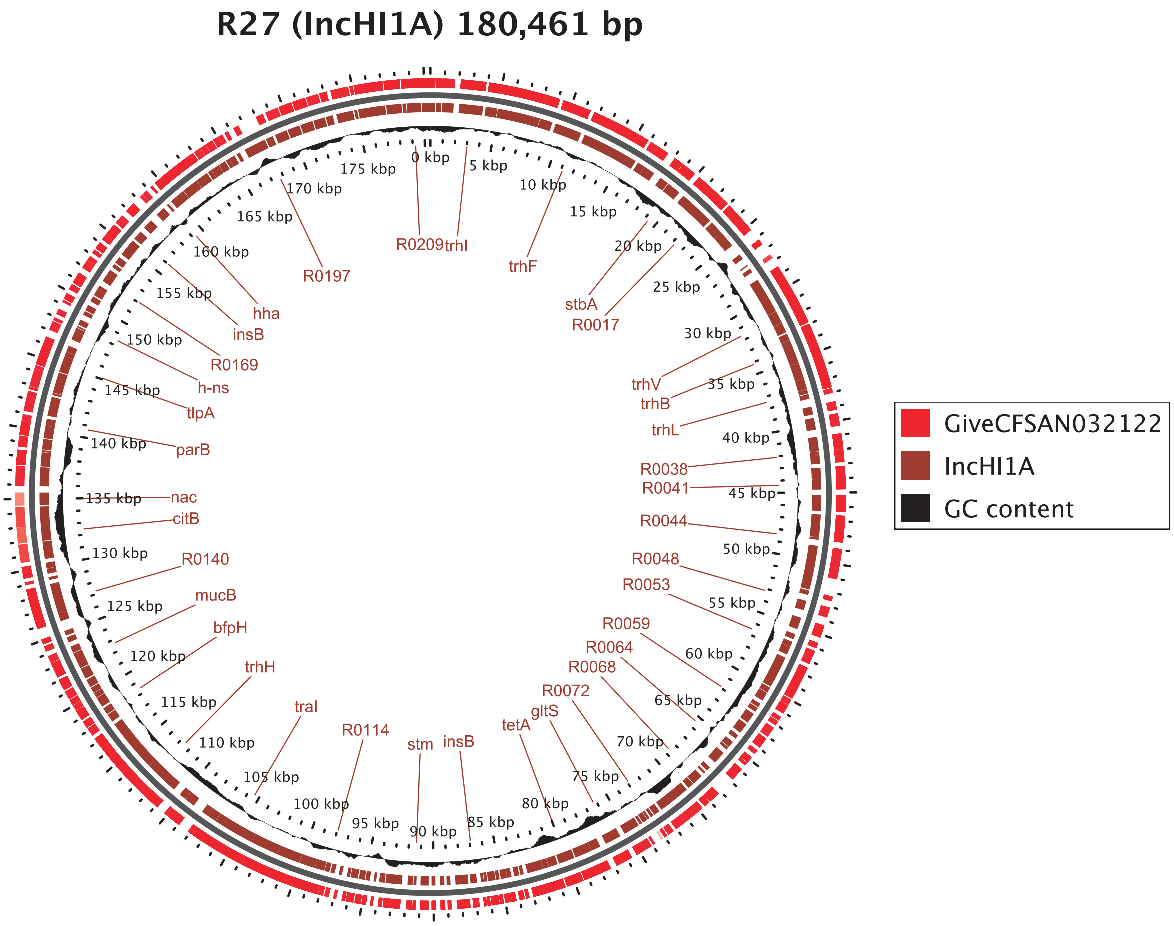

Figure S11. Alignment of reference plasmid R27 (IncHI1A) to the contigs of the newly sequenced *Salmonella* Give isolate (see Table 2 for accession number) where this plasmid was predicted by the PlasmidFinder tool. Map generated with GView, version 1.7 through the tblastx program, with e-value=0.001, alignment cutoff=50, identity cutoff=70, and no filtering of low complexity sequences.
